# A Flow Cytometry–Based Method for Assessing CAR Cell Binding Kinetics Using Stable CAR Jurkat Cells

**DOI:** 10.21769/BioProtoc.5021

**Published:** 2024-06-20

**Authors:** Alex Shepherd, Bigitha Bennychen, Zafer Ahmed, Risini D. Weeratna, Scott McComb

**Affiliations:** 1 Human Health Therapeutics Research Centre, National Research Council, Ottawa, ON, Canada; 2 Centre for Infection, Immunity and Inflammation, University of Ottawa, Ottawa, ON, Canada; 3 Department of Biochemistry, Microbiology, and Immunology, University of Ottawa, Ottawa, ON, Canada

**Keywords:** CAR-T, Jurkat, Screening, T cell, Cellular avidity, High throughput, Flow cytometry, Cell-to-cell interaction, Cellular kinetics

## Abstract

Chimeric antigen receptors (CARs) are synthetic fusion proteins that can reprogram immune cells to target specific antigens. CAR-expressing T cells have emerged as an effective treatment method for hematological cancers; despite this success, the mechanisms and structural properties that govern CAR responses are not fully understood. Here, we provide a simple assay to assess cellular avidity using a standard flow cytometer. This assay measures the interaction kinetics of CAR-expressing T cells and targets antigen-expressing target cells. By co-culturing stably transfected CAR Jurkat cells with target positive and negative cells for short periods of time in a varying effector–target gradient, we were able to observe the formation of CAR-target cell doublets, providing a readout of actively bound cells. When using the optimized protocol reported here, we observed unique cellular binding curves that varied between CAR constructs with differing antigen binding domains. The cellular binding kinetics of unique CARs remained consistent, were dependent on specific target antigen expression, and required active biological signaling. While existing literature is not clear at this time whether higher or lower CAR cell binding is beneficial to CAR therapeutic activity, the application of this simplified protocol for assessing CAR binding could lead to a better understanding of the proximal signaling events that regulate CAR functionality.

Key features

• Determines CAR receptor cellular interaction kinetics using a Jurkat cell model.

• Can be used for a wide variety of CAR target antigens, including both hematological and solid tumor targets.

• Experiments can be performed in under two hours with no staining using a standard flow cytometer.

• Requires stable CAR Jurkat cells and target cells with stable fluorescent marker expression for optimal results.

**Graphical overview**


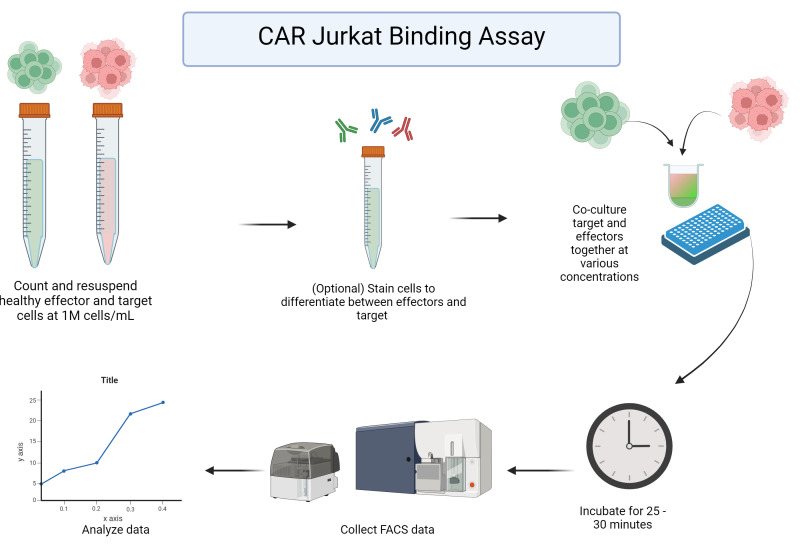


## Background

Chimeric antigen receptor (CAR) T-cell therapy is an engineered cellular cancer therapy that reprograms a patient’s T cells to target a specific protein, mimicking natural T-cell receptor (TCR) function but redirected toward antigens found on the surface of cancer cells. CAR-T cells have emerged as a highly successful tool for providing robust therapeutic responses against refractory or relapsed hematological cancers, with ongoing research across many domains of immunology to extend the range and efficacy of CAR-T treatments for solid tumor malignancies [1–3].

Many advancements have been made in understanding the novel biology of CAR-modified T cells; however, CAR-T-cell screening models still struggle to choose candidate binding elements based on in vitro assessments of CAR-T activation and cytotoxicity assays. Whilst activation markers, such as CD69, are well-documented using CAR-Jurkat and primary CAR-T-cell screening methods, it is unclear how well these markers can predict in vivo efficacy of the CAR-T cells [4,5]. Recently, cellular avidity was recognized as a property of CAR-expressing cells that varies between different CAR constructs and correlates with important functional properties such as CAR expansion, CAR-activated trogocytosis, and CAR-T exhaustion [6–9].

Cellular avidity is defined by the total sum of cell-to-cell interactions, encompassing the whole immunological synapse and many cellular adhesion processes [10]. Given that cell binding is a near-immediate consequence of CAR signaling and proximal T-cell response processes, understanding the nature of this interaction and how it differs between standard TCR interaction vs. CARs is an important area of research. Investigating how cellular avidity changes with different CAR binding domains or structural designs is also vital for improving CAR discovery and development. It is important to note that while the specific properties of an antibody used to create a CAR impact the overall cellular avidity, the affinity and avidity of an antibody are entirely distinct from the complete cell-to-cell interaction that defines “cellular avidity”.

For soluble proteins such as antibodies, avidity is defined as the overall accumulated strength of interaction through multiple interactions, but it can vary widely depending on the nature of the target antigen and the specific conditions of the assay. The measured avidity of an antibody is usually reported as the half-maximal binding concentration observed over a titrated binding assay, this property being referred to as the apparent affinity (K_D_). In contrast to antibodies, using the assay described herein, we have observed that the half-maximal binding of CAR-expressing cells over a titrated range of target cell concentration does not vary between CAR proteins; rather, we find that CAR cells consistently reach a point of saturated maximal binding, wherein a varying proportion of CAR-expressing cells will form a strong bond with target cells (we refer to this as a CAR-target doublet). The strength of this interaction, while not encompassing the entirety of the kinetics, approximates what is commonly called cellular avidity. This observation of a varying binding rate between CAR-expressing and target cells has similarly been reported using an assay based on a resonance force ramp microscopy using the Lumicks zMovi device [6], which directly tests the strength of interaction by attempting to measure the force at which CAR-target pairs are dissociated. We find that the rate of CAR-target doublet formation increases with higher target cell density, eventually reaching a saturation point. Furthermore, we find the rate of doublet formation varies between different CARs [11] or CAR designs [5]. While this assay does not directly test the strength of interaction and therefore is only an approximation of overall cellular avidity, we believe the unique target binding properties for each CAR serve as valuable metrics for CAR screening and assessment. As such, this assay could be used as a replacement to, or in conjunction with, direct avidity assays for optimal CAR selection.

Here, we provide a complete description of our cellular binding assay that serves as a quick, high-throughput addition to CAR-T screening workflows using a standard flow cytometer. Through the quantification of cell/target doublets formed within CAR Jurkat target cell co-cultures over a wide range of effector-to-target ratios, the unique cell binding properties associated with each CAR can be determined. This assay provides consistent results and can be employed with both solid tumors (see validation of protocol) and hematological tumor models.

## Materials and reagents

**Biological materials**

1. Jurkat e6.1 (ATCC, catalog number: TIB 152) expressing a variety of CAR proteins
2. Ramos (ATCC, catalog number: CRL-1596) or other relevant target tumor cell line(s)
  Target cells have been engineered with Nuclight Lenti-Red (Sartorius, Germany; 4476). It is also possible to use non-fluorescent cells for this assay, but antibody prestaining of targets or effectors is recommended as detailed in section B.

**Reagents**

1. Fetal Bovine Serum (FBS) (Sigma Life Sciences, catalog number: F2442-500ML)
2. L-Glutamate (Gibco, catalog number: 25030-081)
3. Penicillin/Streptomycin (Pen/Strep) (Gibco, catalog number: 15140-122)
4. RPMI 1640 (Gibco, catalog number: 21870-076)
5. Dulbecco’s phosphate-buffered saline (DPBS) (Gibco, catalog number: 14190-144)
6. VHH or scFv-specific fluorescent antibody (optional) (generated in-house)
  - An analogous anti-VHH polyclonal product (Jackson Laboratories, catalog number: 128-605-232)
  - For other CARs, various anti-scFv or anti-linker antibodies are available
7. CD45 or other Jurkat/T-cell specific antibody (optional) (BD Pharmingen, catalog number: 560178)
8. CD19 or other target-specific antibody (optional) (BD Horizon, catalog number: 612938)

**Solutions**

1. R10 complete (see Recipes)

**Recipes**

1. **R10 complete**
  **Table:** 

Reagent	Final concentration	Quantity or Volume
RPMI 1640		500 mL
FBS	100 mL/L	50 mL
L-Glutamate	10 mL/L	5 mL
Pen/strep	10 mL/L	5 mL

**Laboratory supplies**

1. 96-well U-bottom plate (Falcon, catalog number: 353077)

## Equipment

1. BD LSRFortessa flow cytometer
2. BD LSRFortessa flow cytometer HTS plate reader

## Software and datasets

1. FACS Diva
2. FlowJo
3. GraphPad Prism 10

## Procedure

**Figure 1.**
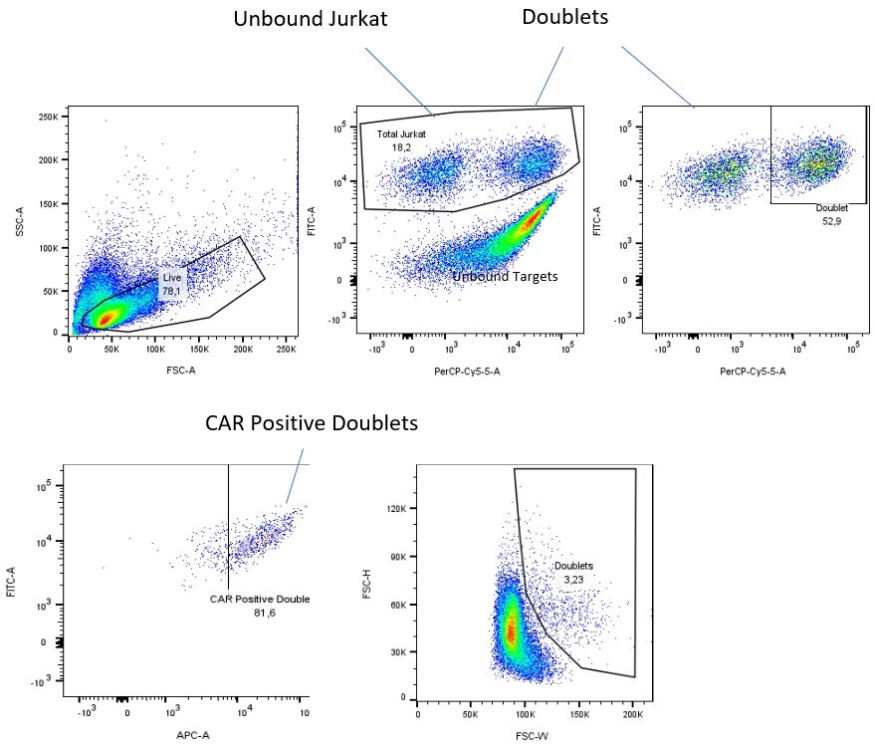
Gating method for fluorescently labeled and sorted cells. Dead cells and debris are gated out (top left), then unbound targets are removed (top middle). Finally, doublets are separated from unbound chimeric antigen receptor (CAR) Jurkat cells and displayed as a percentage of parent (top right). CAR confirmation of doublets can be confirmed using a CAR-specific surface stain (APC channel here), removing auto-fluorescent false positives from doublets that are dead cells or cellular debris (bottom, left). An alternative method of analyzing doublets using their increased size on forward scatter (bottom, middle). This method will require some additional calculations to find the number of living Jurkat cells.

- **Stable CAR Jurkat preparation**
*Notes:*
*1) This assay has been optimized to use both target and effector cells to sort for 100% CAR or reporter-positive cells. Doing so will significantly improve data analysis and data quality.*
*2) We also recommend testing any cells being used for mycoplasma before use in this assay.*
*3) CAR Jurkat cells and target cells should be in healthy log-phase growth conditions prior to initiating the avidity assay, as described below. This can be accomplished by splitting cells 1–2 days before use.*
*4) If using adherent cells, aim to have cells with a confluency of 70%–80%.*
*5) Jurkat cells can be visually assessed for cell health (healthy Jurkat cells will be round and form small clumps) alongside a viability stain when counting. Do not use cells that are less than 80% viable. We have found that cell health can have a major impact on the consistency of results for this assay.*
  1. Cells used to make stable CAR Jurkat or Nuclight target cells should be healthy, log-phase cells prior to lentivirus exposure. Lentiviral transduction should only be performed by trained professionals and handled with all safety protocols in mind. A full lentivirus creation and transduction protocol can be found in Tandon et al. [12]. Please note that all work using active lentivirus requires BSL2+ safety and training.
    While details are not provided here, a general workflow for generating stable CAR Jurkat cells involves the following steps: CAR-lentiviral particle production as per linked protocol; (optional) lentiviral concentration using high-speed centrifugation; transduction of Jurkat cells with CAR-lentiviral particles; cell sorting to isolate a pure population of CAR Jurkat cells; and (optional) cryopreservation of the CAR Jurkat cell line for various downstream analyses, such as the one described here.
  2. Once transduced cells are viral vector–free (typically after three media changes and at least 7 days at 37 °C), cells can be safely sorted for 100% CAR or fluorescent marker expression for best results. To generate target cells with stable Nuclight-Lenti (Sartorius, USA) expression, puromycin selection can be used. For stable cells with no resistance genes, the best option is the derivation of single-cell clone populations using cell sorting or limiting dilutions.
- **CAR Jurkat binding assay plate setup**
  1. Remove your target and stable CAR Jurkat cells from the incubator and count them, ensuring cells have at least 80% viability and are in the log phase of growth. We recommend not using cells that have been in culture for more than three months.
  2. Spin down and bring CAR Jurkat and target cells to 1 million cells/mL in separate suspensions in R10 complete media. To enhance discrimination between effector cells, target cells, or doublets, you may use prestaining with antibodies that are specific to effector cells (e.g., CD45 or anti-VHH/scFv) or target cells (e.g., CD19). This is especially helpful if you are not using cells with stable fluorescent protein expression, as shown in Figure 1. If you are using an antibody stain for your CAR or target cells, spin down and stain your cells now.
    - For staining, resuspend the cell pellet in 100 µL of R10 complete medium, stain, and leave in the dark at room temperate for 15 min before washing off the excess stain. To wash, add 5 mL of 1× PBS and spin down the cells at 500× *g* for 3 min before removing the PBS and resuspending the cells in R10 complete medium, bringing the cell concentration back up to 1 M/mL.
    - Exemplary data provided here uses the Ramos human lymphoma cell line as the target cell line, although this assay can be successfully performed with a wide variety of adherent and non-adherent target cells.
  3. Add 50 µL of R10 complete medium to each well of your 96-well plate.
  4. Add target cells to each well at the ratio described in Table 1 below. An example plate map is also given in Table 2.
    **Table 1.** Effector-to-target dilution chart

Ratio	# of cells (effector/target)	Vol. needed at 1 M cell/mL effector (µL)	Vol. needed at 1 M cell/mL target (µL)	Row letter
1:10	5,000/45,000	5	45	A
1:5	10,000/40,000	10	40	B
1:2	15,000/30,000	15	30	C
1:1	25,000/25,000	25	25	D
2:1	30,000/15,000	30	15	E
5:1	40,000/10,000	40	10	F
10:1	45,000/5,000	45	5	G
    **Table 2.** Example plate map

E:T		**CAR Samples**											
		CAR1		CAR2		CAR3		CAR4		CAR5		Control (irrelevant CAR)	
1:10												
1:5												
1:2												
1:1												
2:1												
5:1												
10:1												
No target
  5. Plate Jurkat cells at the appropriate concentration as shown above. If done correctly, all wells should contain 100 µL of sample. Manually and gently shake the plate from side to side.
  6. Place the 96-well plate in an incubator at 37 °C and 5% CO_2_ for 30 min.
    - If desired, this assay can be run at 4 °C as a control. Doublets should not form at this temperature.
    - This assay has been performed in as little as 10 min and as long as 4 h. Thirty minutes is our recommended minimum time for best results. It should be noted that, if this assay is being performed with an adherent target cell line, the incubation should not last long enough for cells to adhere.
- **CAR Jurkat avidity flow cytometry**
  1. During the plate incubation, start up the flow cytometer and perform Cytometer Setup and Tracking (CS&T) and a system prime of the high-throughput sampler (HTS) to ensure the flow cytometer is functioning properly.
  2. Once the incubation period has elapsed, run the plate on the flow.
    - This assay is optimized for an HTS setup for the BD LSRFortessa. Depending on your equipment, some modifications may be necessary.
    - While some settings may vary, those used for our setup are as follows:
      1) Samples were run on a 96-well U-bottom plate, collecting 75 µL of sample or 50,000 events per well.
      2) Cells were mixed by the cytometer and collected at a rate of 3 µL/s.
      The relevant voltage settings are as follows:
      a) FSC 180 V
      b) SSC 250 V
      c) FITC 480 V
      d) PerCP-Cy5-5 730 V
      e) APC 600 V

## Data analysis

Data analysis of the raw flow data should be performed in the latest version of FlowJo but could be similarly performed with alternative flow cytometry analysis software. To start, gate out dead cells or cellular debris using forward and side scatter (Figure 1, top left). From here, analysis can be performed in two separate ways to obtain cellular avidity reading. If you are using stained cells or cell lines with a reporter, set your laser reading to display your cellular stains for the CAR and your target cell line with acceptable signal strength. The example shown below uses stable fluorescent markers: NeonGreen (FITC) for the CAR and Nuclight Red (or mKate2; PerCP Cy5.5) for the target cell lines. Should the cells bind, this should show three or four separate populations: unbound CAR Jurkat cells, unbound target cells, and bound doublets that express both the CAR and target colors (Figure 1, top middle). If your stable population of CAR Jurkat is unsorted, you may see an unstained population of non-CAR expressing Jurkats as well.

If you do not have stains in your CAR or target cells, instead display the forward scatter height by the forward scatter width. Cell doublets present in the well should be wider than the rest of the cells and can be selected alongside the other unbound Jurkats to give a similar result (see Figure 1, bottom middle). It should be noted that if the target cells are significantly bigger than the Jurkat cells, this method may not work as well, so we recommend using a staining method if possible.

To generate a CAR:target curve, use flow cytometry analysis software to exclude unbound target cells and then record your doublet population as a percentage of parents of the entire Jurkat population in the well (Figure 1, right). This will give the percentage of the CAR Jurkat population bound at that time and ratio. The ratio of bound doublet cells to free CAR Jurkat can be calculated manually as follows:

$\frac{Number\;of\;CAR–positive\;bound\;cells}{Total\;numbler\;of\;living\;CAR-Jurkat\;cells}\;$ ×  100%

**Figure 2.**
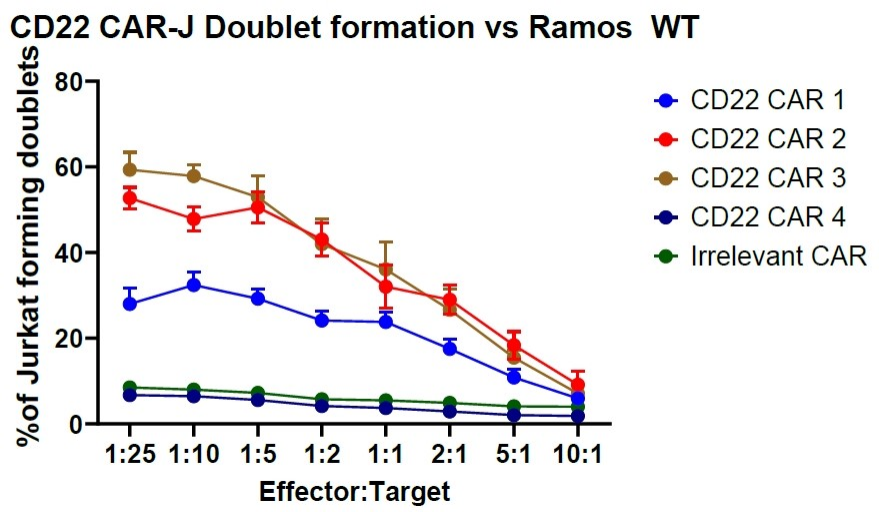
Exemplary data of CD22 chimeric antigen receptor (CAR) Jurkat binding kinetics against Ramos WT cells. The data displayed was performed in triplicate and displays data plus standard error of mean (SEM). Note that the “Irrelevant CAR” data set (green) has been nudged up two data points for visibility. Exported data was imported into PRISM 10 and graphed in the “grouped” format.

This data can be graphed using appropriate data visualization software such as GraphPad PRISM 10, resulting in a curve with maximum binding occurring in an excess of target cells (>1:10–1:25 effector:target ratio) (see Figure 2).

## Validation of protocol

This protocol or parts of it have been used and validated in the following research articles:

McComb et al. [5]. Programmable attenuation of antigenic sensitivity for a nanobody-based EGFR chimeric antigen receptor through hinge domain truncation. *Frontiers in Immunology.* 13. (Figure 4, Supplementary Figure 4)

McComb et al. [11]. Discovery and Pre-Clinical Development of a Therapeutically Active Nanobody-based Chimeric Antigen Receptor targeting human CD22. *Molecular Therapy Oncology*. 32, 1, 200775. (Figure 2F)

## General notes and troubleshooting

**General notes**

1. As mentioned above, this assay is biological in nature and requires active cellular signaling to induce cell-to-cell interaction. No cell-to-cell binding will occur at 4 °C, as signaling processes are inactive at this temperature. This can be used to test for non-specific binding and establish a background (Figure 3). Additionally, we recommend running the assay in parallel with a negative cell line and a non-specific/irrelevant specificity CAR to establish non-specific binding control.
  **Figure 3.**
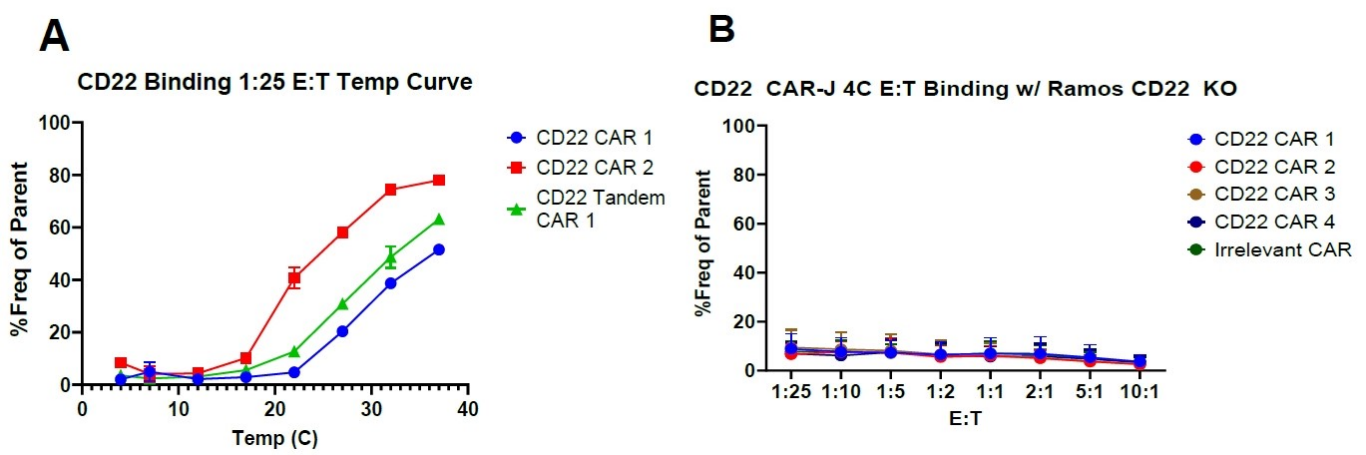
Chimeric antigen receptor (CAR)-Jurkat binding assessment using a temperature curve. (A) CD22 CAR-J cells were run first at 1:25 effector:target starting at 4 °C and incrementing the temperature gradually until 37 °C. Then, the binding assay was repeated in full at 4 °C. CAR-J cells cannot bind targets at 4 °C and only begin to show binding at approximately 21 °C. (B) The standard assay described above performed with target antigen-negative cells. Experimental data was performed in triplicate. Error bars show standard error of mean (SEM).
2. This assay is not CAR exclusive. This can work with any biological that causes T cells to bind, such as bispecific T cell engagers (BiTEs), that cause cell binding and T-cell synapse formation.
3. We are currently working on a version of this assay using lentiviral-transduced T cells. The primary cell version can be successful, but the nature of expanded donor T cells requires additional attention to experimental details to yield consistent results. Specifically, the proliferation state, transduction rate, and donor source are all key variables that can interfere with the measurement of CAR cellular avidity. Thus, we feel that this assay is best measured using Jurkat T cells for several reasons: (1) Jurkat cells lack a cytotoxic response and have limited cytokine activity, with no risk of the bound cell dying before passing through the flow cytometer; (2) Jurkat CAR cells can be sorted to 100% CAR cells without issues due to their steady proliferation; (3) Jurkat cells maintain a consistent differentiation profile, while primary T cells can vary widely throughout manipulations. For these reasons, we recommend using Jurkat cells for performing this assay.

**Troubleshooting**

Problem 1: Cells do not form doublets in co-culture within 30 min.

Possible cause: Wrong culture plate used.

Solution: Change the assay plate to a 96-well treated U-bottom plate. This assay is optimized for U-bottom 96-well plates. Attempts in larger wells or wells with flat bottoms generally result in sub-optimal results.

Possible cause: Cells are unhealthy.

Solution: Re-attempt assay with cells in log-phase of growth. Cell health has been shown to have a direct impact on the speed and count of doublets in solution.

Problem 2: Cells do not form doublets ever.

Possible cause: Incubation is performed in untreated plastic or the wrong plastic type.

Solution: Ensure the assay is performed in a 96-well plate made of non-pyrogenic vacuum gas plasma-treated polystyrene. Incubations done in polypropylene tubes such as PCR tubes or 1.5 mL Eppendorf or in untreated polystyrene have been unsuccessful, as it appears the cells adhere to the plastic and have difficulty binding.

## Supplementary information

The following supporting information can be downloaded here:

1. Figure S1. Cytometer and HTS setup.
